# Revisiting Oxygen Transport Features of Hemocyanin with NEVPT2 Level QM/MM Calculations

**DOI:** 10.1021/acs.jctc.4c01668

**Published:** 2025-02-06

**Authors:** Francesca Fasulo, Aarón Terán, Michele Pavone, Ana B. Muñoz-García

**Affiliations:** † Department of Physics “E. Pancini”, University of Naples Federico II, 80126 Napoli, Italy; ‡ Department of Chemical Sciences, University of Naples Federico II, 80126 Napoli, Italy

## Abstract

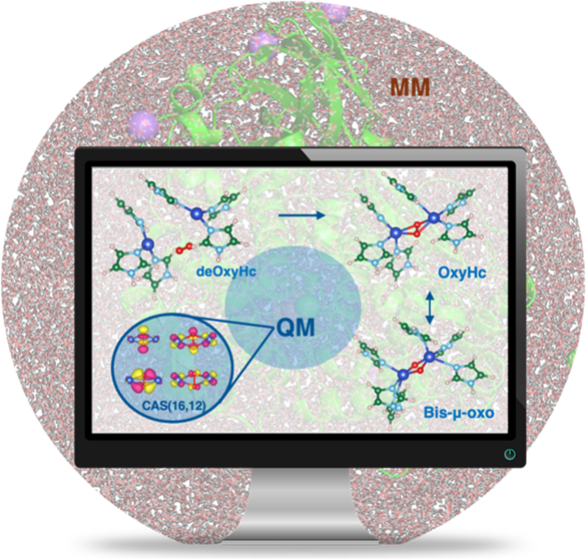

This study explores the oxygen-binding mechanism and the potential peroxo-to-bis-μ-oxo isomerization in hemocyanin (Hc) using a quantum mechanics/molecular mechanics (QM/MM) approach at the multireference NEVPT2 level of theory (QM[NEVPT2]/MM). Our results support the previously proposed mechanism for Hc oxygen binding, involving two nearly simultaneous electron-transfer (ET) steps and a triplet–singlet intersystem crossing (ISC). However, we find that the first ET step occurs prior to ISC, resulting in the formation of a stable singlet superoxide intermediate through a low-energy barrier. The second ET leads to the formation of a singlet oxy-hemocyanin species featuring the characteristic peroxo–Cu_2_O_2_ “butterfly” core. Moreover, QM[NEVPT2]/MM simulations reveal a lower-energy barrier for the peroxo-to-bis-μ-oxo isomerization compared with density functional theory (DFT), although the peroxo form remains energetically favored within the protein environment. These findings offer new insights into the behavior of the hemocyanin active site, highlighting the importance of considering both the electronic correlation and the protein environment in accurately modeling copper–oxygen interactions in biological systems.

## Introduction

Copper–bimetallic enzymes, such as hemocyanin, tyrosinase, and catechol oxidase, have attracted great interest due to their pivotal roles in biological and catalytic processes, including oxygen transport, catalysis, and oxidative chemistry.^1−4^ In particular, hemocyanin (Hc), the oxygen transport protein in arthropods and mollusks, has been extensively studied due to its interesting active site and reversible oxygen-binding mechanism.^1,4−9^

**Scheme 1:**
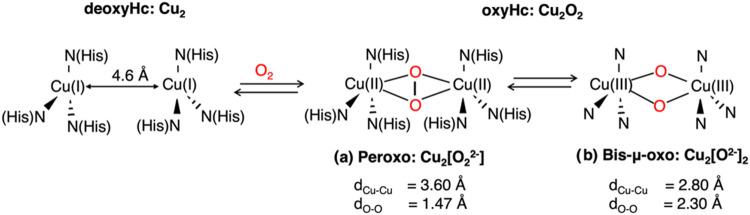
Schematic Representation of the deoxyHc and oxyHc from *Limulus polyphemus* ^5,6,11^ Both possible isomers of oxyHc are illustrated: (a) peroxo (Cu_2_[O_2_^2–^])^5,6^ and (b) bis-μ-oxo forms (Cu_2_[O^2–^]_2_).^11^ Experimental Cu–Cu and O–O bond lengths (d_Cu–Cu_, d_O–O_) are reported.^5,6,11^

Hc active site consists of a dinuclear copper center, surrounded by six histidine residues, which alternates between two states: the deoxygenated form (deoxyHc: Cu_2_), where the copper ions are in the Cu(I) oxidation state, and the oxygenated peroxo-like form (oxyHc: Cu_2_O_2_), where both copper ions are in the Cu(II) configuration. Notably, some synthetic complexes with a Cu_2_[O_2_^2–^] core can undergo isomerization to a bis-μ-oxo (Cu_2_[O^2–^]_2_) configuration^10−13^ (Scheme 1). Crystallographic studies of subunit II in *Limulus polyphemus* have revealed that the Cu–Cu distance in the active site shortens by approximately 1 Å after O_2_ binding, from 4.6 ± 0.2 Å in deoxyHc to 3.6 ± 0.2 Å in oxyHc, while other geometric parameters remain unchanged.^5,6^ This transition allows hemocyanin to bind and release molecular oxygen, enabling the transport to arthropod tissues.

Much effort has been focused on describing the reactivity of this active site. Different synthetic models have been obtained to understand the nature of the Cu–O_2_ bond and the characterization of possible intermediates to provide mechanistic insights into the reaction coordinates. Experimental studies reveal that the binding of triplet O_2_ to the two Cu(I) ions in deoxyHc requires two-electron-transfer (ET) steps, followed by a triplet–singlet intersystem crossing (ISC), which ultimately yields a peroxo-like Cu_2_O_2_ complex in the oxyHc state.^14,15^ Indeed, the dicopper center in oxyHc is diamagnetic, exhibiting a strong antiferromagnetic interaction (−2*J* > 600 cm^–1^).^16−18^ Several theoretical investigations, including density functional theory (DFT) and multireference methods, have addressed these phenomena.^3,7−9,19−25^ Solomon et al.^9^ explored these processes with small model systems to mimic the Hc active site at the DFT level of theory, and they predicted a simultaneous two-electron transfer to give a peroxo-like Cu_2_O_2_ complex, followed by the ISC. However, the computed thermodynamics were found to be highly dependent on the size of the model systems and DFT calculations predicted a planar Cu_2_O_2_ core for the oxyHc state instead of the butterfly-like moiety observed in the crystal structure of hemocyanin.^5−8,22^ Previous ab-initio investigation also pointed out that the stability of the singlet over the triplet state in oxygenated copper dimers is significantly influenced by the copper ligands, which modulate the electronic structure and energetic landscape of the system.^19^ Although small models shed light on the essential copper–oxygen chemistry, they do not fully capture the intricate effects of the protein environment on the reaction mechanisms.

To overcome these limitations, Saito et al.^24^ proposed a quantum mechanics/molecular mechanics (QM/MM) approach, integrating the accuracy of quantum mechanical calculations with the comprehensive modeling of the protein environment through molecular mechanics.^26−32^ Using DFT for the QM region in a QM[DFT]/MM calculation, they found that the initial ET step results in a nonbridged superoxo intermediate with a low-energy barrier, with the second ET step proceeding after a triplet–singlet intersystem crossing (ISC) with an even lower barrier, forming a side-on oxyHc complex with the characteristic Cu_2_O_2_ butterfly core. Indeed, the low barriers for both steps suggest that the two ET processes are expected to occur very rapidly and nearly simultaneously. Although their findings align with the experimentally proposed mechanism of a nearly synchronous two-electron transfer,^14,15^ the calculated binding energy (∼−18.9 kcal/mol) exceeds the experimentally obtained binding enthalpy for hemocyanin and analogous synthetic model complexes, which fall between the −14 and −6.0 kcal/mol range.^33,34^ This discrepancy underscores the challenges of accurately modeling the energetics of oxygen binding in Hc, even when including the realistic environment of the protein in the calculation.

A primary challenge in accurately modeling the oxygen-binding process in Hc lies in the complex multireference character of the Cu_2_O_2_ core. This electronic feature demands advanced post-Hartree–Fock methods, such as the Complete Active Space Perturbation Theory (CASPT2)^35^ or the similar n-electron valence-state second-order perturbation theory (NEVPT2).^36^ Such multireference methods should provide accurate energetics wherever spin-unrestricted DFT (UDFT) highly sensitive to the functional choice fails, but requires large active spaces and significant computational resources, often limiting the system size.

In addition to the electronic structural challenges for the Hc oxygen-binding process, the geometric flexibility of the Cu_2_O_2_ core raises additional questions, as the core adopts multiple isomeric forms including the side-on peroxo and bis-μ-oxo isomers, both of which have been observed in synthetic model complexes of hemocyanin and related enzymes like tyrosinase.^11−13^ Tolman et al.^13^ discovered that synthetic complexes (L-Cu_2_O_2_) can reversibly bind dioxygen and undergo isomerization, involving the O–O bond cleavage from a peroxo (Cu_2_[O_2_^2–^]) to a bis-μ-oxo form (Cu_2_[O^2–^]_2_) configuration (Scheme 1). The bis-μ-oxo form features a shorter Cu–Cu distance of approximately 2.8 Å and a longer ∼2.3 Å. While several L-Cu_2_O_2_ complexes share similar structural, magnetic, and spectroscopic features, they exhibit distinct reactivities due to the peroxo-to-bis-μ-oxo isomerization.^37−39^ In Hc, the bis-μ-oxo form could be critical for the reversible binding of oxygen and for deactivation of reactive oxygen species (ROS), as suggested by some experiments.^13,40−44^ Therefore, the isomerization between these two forms has attracted significant attention, particularly regarding the activation of the O_2_ and the cleavage of the O–O bond cleavage.

DFT studies on L-Cu_2_O_2_ complexes predict peroxo as the more stable form, while multireference investigations showed an energetically favored bis-μ-oxo isomer and flat potential energy surface for Cu_2_O_2_ isomerization, especially when dynamic electron correlation is taken into account.^12,13,23,45−51^ In fact, the dynamic interaction between the Cu 3*d* and the O_2_ π* orbitals plays a crucial role in stabilizing the bis-μ-oxo structure. Several studies have highlighted that the O–O bond cleavage in this configuration enhances the multiconfigurational character of the electronic wave function, thereby rendering the bis-μ-oxo form energetically more favorable.^45,48−50^ These theoretical models applied to the peroxo-to-bis-μ-oxo isomerization, however, neglect the role of the protein–ligand interactions, which significantly modulate the degree of charge transfer from Cu(II) to O_2_^2–^, as well as the covalency of the Cu–O bonds and O–O elongations.^23,37,48^

In this study, we revisit the oxygen-binding mechanism and the peroxo-to-bis-μ-oxo isomerization in Hc using state-of-the-art QM/MM approaches at the multireference NEVPT2 level of theory (QM[NEVPT2]/MM). Although DFT successfully predicts the feasibility of oxygen binding, it often fails to accurately describe the electronic structure of systems with highly correlated electrons, such as radical species (e.g., superoxide ion O_2_^–^) and mixed Cu_2_ oxidation states. In contrast, multireference methods like NEVPT2 effectively capture the electronic structure of these challenging species by accounting for both static and dynamic correlation effects. However, the significant computational cost of these methods typically restricts their application to small systems. By integrating multireference approaches within a QM/MM framework, we aim to provide a holistic description of the reactivity of the hemocyanin active site toward molecular oxygen, capturing both the electronic complexity and the influence of the protein environment.

Concerning the oxygen binding, our results show that the first ET step is followed by a nearly simultaneous triplet–singlet ISC leading to a stable singlet Cu_2_–O_2_^–^ intermediate with a low-energy barrier. The second ET yields a singlet oxyHc species with the characteristic peroxo–Cu_2_O_2_ butterfly core. Our QM[NEVPT2]/MM simulations reveal a lower-energy barrier for the peroxo-to-bis-μ-oxo isomerization than DFT. However, in contrast to previous studies on L-Cu_2_O_2_ complexes,^12,13,23,45−51^ the peroxo form is energetically preferred in the protein environment at this level of theory. Additionally, we confirm the pronounced multireference character of the bis-μ-oxo isomer, which primarily exhibits a peroxide nature (Cu^2+^)_2_(O_2_^2–^), 63%, and only a 4% oxide character ((Cu^3+^)_2_(O^2–^)_2_). Overall, our findings enhance the understanding of the Hc active site and highlight how crucial it is to consider both protein environments and electronic correlation in order to effectively describe copper–oxygen interactions in biological systems.

## Methods and Computational Details

**Figure 1:**
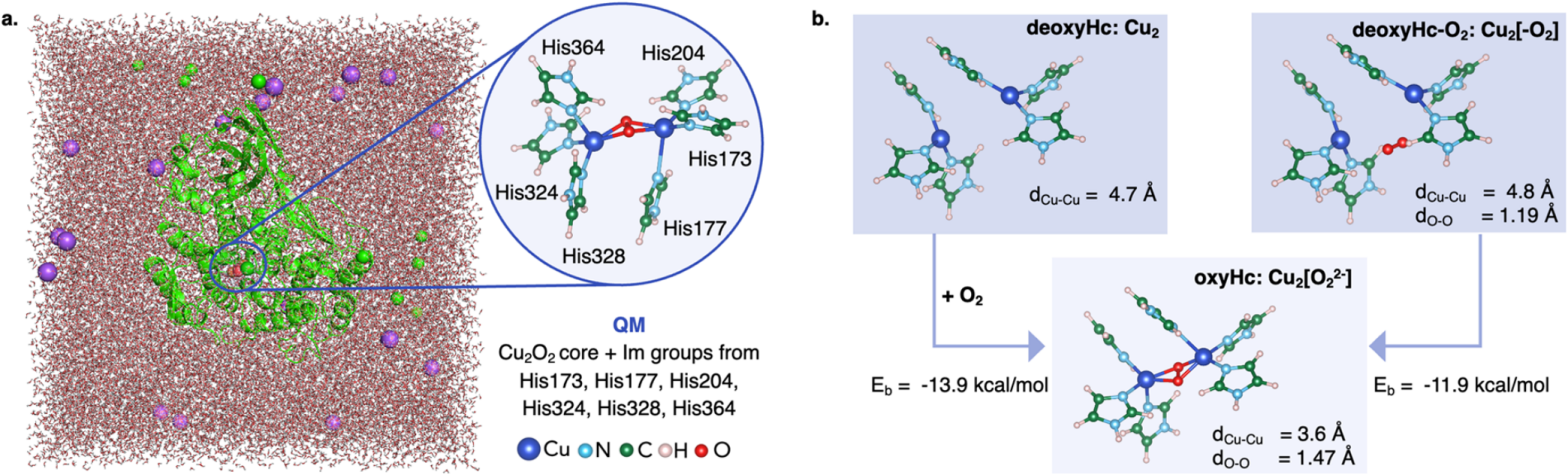
(a) Illustration of the QM/MM model of oxyHc protein in a 50 Å cubic box filled with water molecules and Na^+^ and Cl^–^ ions. The QM region included the Cu_2_O_2_ core and the imidazole groups from the coordinating histidine residues (His173, His177, His204, His324, His328, and His364). (b) Minimum energy structures at the QM[M06-2x]/MM level of theory of the fully deoxygenated Cu_2_ core (deoxyHc: Cu_2_), Cu_2_O_2_ configuration with farther O_2_ (deoxyHc-O_2_: Cu_2_[-O_2_]), and oxygenated Hc (oxyHc: Cu_2_[O_2_^2–^]). Oxygen-binding energies at the QM[M06-2x]/MM level of theory are also reported. Atom color codes: Cu (blue), O (red), C (green), N (light blue), and H (light pink).

The initial structure of oxyHc was taken from *Limulus polyphemus* X-ray crystallographic data (PDB code: 1OXY, resolution 2.4 Å).^6^ Missing residues (18–32, 132–150, 421–427, 522–530, 570–573, and 628) were added using MODELER.^52^ Protonation states of titratable residues (histidine, glutamate, and aspartate) were assigned according to previous work by Saito et al.^24^ Copper Lennard-Jones parameters were adapted from Ungar et al.,^53^ and hydrogen atoms were added using Gromacs.^54^ The system was solvated in a 50 Å cubic box filled with TIP3P water molecules (35270 in total), while any water molecules overlapping the protein were removed. To neutralize the system, a total charge of −7e, 29 water molecules were replaced by 18 Na^+^ ions and 11 Cl^–^ ions, ensuring a minimum distance of 5.5 Å from any protein atoms. A 500 ps molecular dynamics (MD) equilibration run at 300 K was conducted with a 1 fs time step, followed by energy minimization using the CHARMM36 force field^55^ as implemented in Gromacs.^54^ Then, the final snapshot from the MD simulation was used as the starting geometry for the QM/MM optimizations. The QM region included the Cu_2_O_2_ core and the imidazole groups from the coordinating histidine residues (His173, His177, His204, His324, His328, and His364), as illustrated in Figure 1a. QM/MM optimizations were performed using the ASH code^30,56^ interfaced with Gromacs^54^ and the ORCA code.^57^ The def2-TZVP basis set^58^ was employed for all atoms in the QM region. We investigated the oxygen binding and the peroxo-to-bis-μ-oxo isomerization comparing broken-symmetry DFT at the M06-2X level of theory (QM[M06-2X]/MM)^59^ and the NEVPT2 approach (QM[NEVPT2]/MM).^36^ NEVPT2 was chosen due to its proven accuracy in modeling oxygen binding to metal centers^60^ and isomerization processes of dicopper complexes.^46^ Entropy effects are not included in our predictions because experimental findings highlight only a small entropy contribution at room temperature (an average of ∼3 cal/K·mol),^9,33^ mostly due to protein structural fluctuations. We analyze the Hirshfeld population analysis of molecular orbitals (MOs) in the active spaces as implemented in Multiwfn.^61,62^

## Results and Discussion

### QM/MM Energy Landscape of Oxygen Binding at Hc-Cu_2_ Core

As a first step, we optimized the geometry of three Hc models at the QM[M06-2X]/MM level of theory: oxyHc (featuring a bound Cu_2_O_2_ core), deoxyHc (featuring a bare, fully deoxygenated Cu_2_ core), and what we call deoxyHc-O_2_ (i.e. a Cu_2_O_2_ configuration where the O_2_ molecule is 3 Å far away from the active center). Whether the latter model deoxyHc-O_2_, instrumental for NEVPT2 calculations presented below, is a reliable model for fully deoxyHc is validated in the following. The ground state of deoxyHc exhibits a singlet multiplicity arising from the *d*^10^ closed-shell nature of Cu(I) atoms in the absence of O_2_. The deoxyHc-O_2_ species shows a triplet multiplicity, with an unpaired electron on oxygen atoms, as expected from the triplet ground state of the isolated O_2_ molecule (see spin density in Figure S1, SI). When comparing this deoxyHc-O_2_ to the deoxyHc (Figure 1b), there is only a slight elongation of the Cu–Cu distance of 0.10 Å. Both Cu–Cu distances (4.8 Å/4.7 Å), as well as that obtained for oxyHc (3.6 Å), show good agreement with experimental data.^5,6^ The O–O bond lengths in the models including O_2_ match those expected for the peroxide moiety (1.47 Å) in oxyHc and for the free oxygen molecule (1.19 Å) in deoxyHc-O_2_. Computed binding energies are also consistent between deoxyHc (from isolated O_2_, −13.8 kcal/mol) and deoxyHc-O_2_ (from further O_2_, −11.9 kcal/mol) and align well with previous results.^24^ Overall, such information validates our QM[DFT]/MM approach to capture the structural features and energetics of Hc oxygen binding and the deoxyHc-O_2_ as a valid model for mimicking the fully deoxygenated deoxyHc state. Considering these findings, we apply the QM[NEVPT2]/MM calculations to accurately capture the electronic structure of highly correlated systems, such as radical species (e.g., O_2_^–^) and mixed Cu_2_ oxidation states, which can be involved in the Hc oxygen binding.

Validation of the NEVPT2 method requires careful definition of the system in terms of the number of electrons and molecular orbitals (MOs), termed the complete active space (CAS) within the CASSCF approach. Based on previous studies,^19,46,60,63^ we expanded the active space from CAS(6e,4o) to CAS(16e,12o), given by all of the O_2_ MOs (s_s_ s_s_* s_pz_ p_px_ p_py_ p_px_* p_py_* s_p_*) and two *d*_*xz*_ Cu orbitals for the oxyHc system (see Figure S2, SI). With the larger active space (CAS(16e,12o)), we compute a ground-state singlet–triplet splitting (−2*J* = 690 cm^–1^; see Table S1, SI), which is consistent with the experimentally observed diamagnetism (−2*J* > 600 cm^–1^) in oxyHc.^16−18^ Thus, we rely on this active space for the accurate description of oxygenated species and adopt it for all subsequent QM[NEVPT2]/MM investigations.

**Figure 2:**
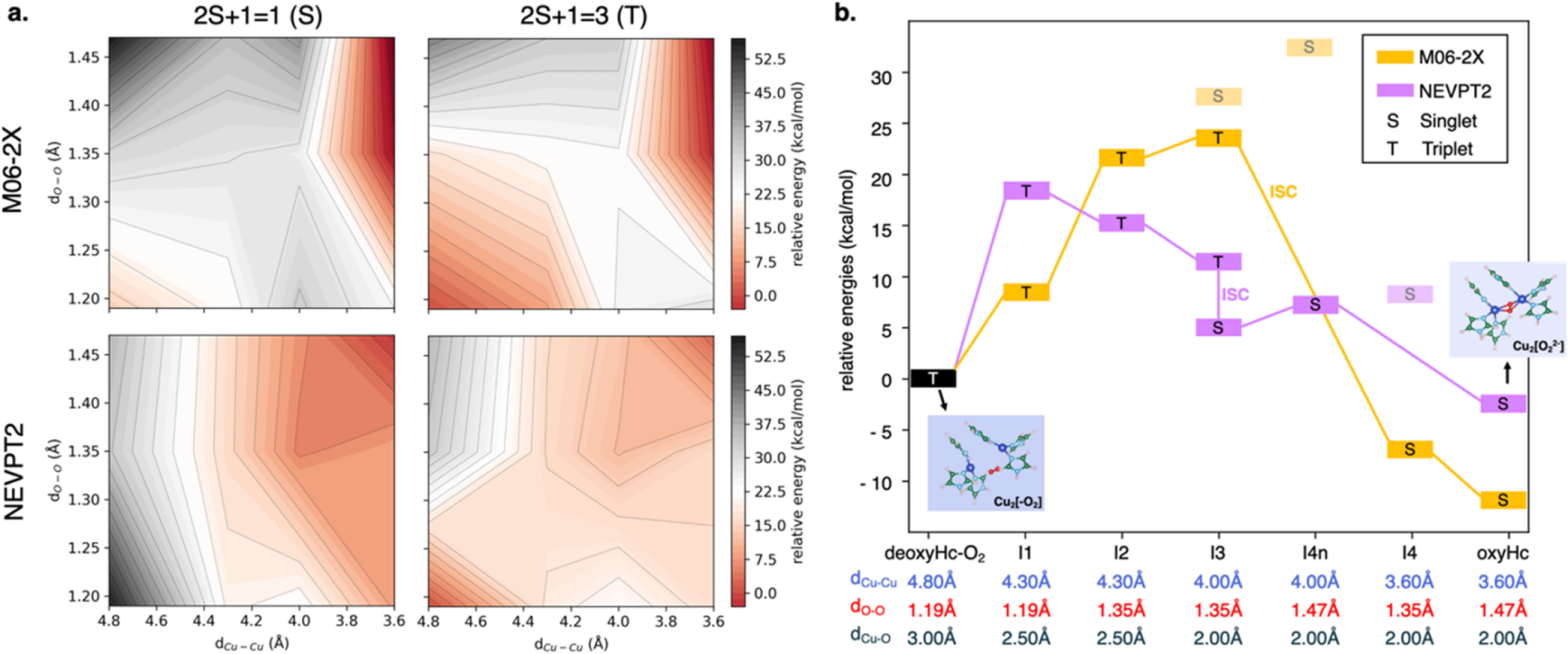
(a) Color energy maps of oxygen binding at the Hc active site computed at the QM[M06-2X]/MM and QM[NEVPT2]/MM level of theory. (b) Energetics of oxygen binding at the Hc active site computed at the QM[M06-2X]/MM (orange line) and QM[NEVPT2]/MM (violet line) levels of theory. Spin multiplicity (S/T) and the scan variables (d_Cu–Cu_, d_O–O_, and d_Cu–O_) are declared. Minimum energy structures of deoxyHc-O_2_ and oxyHc are shown, while for the intermediate states (IN, *N* = 1, 2, 3, 4*n*, 4), see Figure S3b, SI. Relative energies are referred to as deoxyHc (*E*_T(4.8, 1.19)_).

Inspired by previous works,^9,19^ we address the oxygen binding at the Hc active site (Cu_2_) via a potential energy surface (PES) scan in two coordinates: (i) the oxygen–oxygen bond length (d_O–O_) and (ii) the Cu–Cu distances (d_Cu–Cu_). The two PESs for deoxyHc and oxyHc are generated by considering the dioxygen bond length of the typical oxygen molecule (d_O–O_ O_2_ = 1.19 Å), superoxide bond lengths (d_O–O_ O_2_^–^ = 1.35 Å), and the maximum value of the peroxide moiety (O_2_^2–^) in oxyHc (d_O–O_ O_2_^2–^ = 1.47 Å), while we explored the Cu–Cu distance between 4.8 Å (deoxyHc) and 3.6 Å (oxyHc), specifically d_Cu–Cu_ = (4.8, 4.3, 4.0, 3.6 Å). For each combination of (d_Cu–Cu_, d_O–O_), we optimized the distances between the oxygen molecule and the Cu atoms (d_Cu–O_) while also evaluating three fixed d_Cu–O_ (2.0, 2.5, 3.0 Å). Since the minimum energy structures align with the minima from the d_Cu–O_ scan, we considered the different d_Cu–O_ values also for the multireference approach. Separated scans have been performed for the singlet (S) and the triplet (T) states. Accordingly, any point along the corresponding PES is labeled as S/T-(d_Cu–Cu_, d_O–O_), indicating the singlet/triplet configuration of a given structure featuring the (d_Cu–Cu_, d_O–O_) pair of coordinates. Figure 2a displays the color energy maps for Hc oxygen binding at the QM[NEVPT2]/MM level of theory, compared to those delivered by QM[M06-2X]/MM. Since both methods predict the same trend for the d_Cu–O_, only the minima of this variable are reported in the PES. These energetic landscapes may be modulated by water molecules close to the active site. Due to the complexity and dynamic nature of water–copper interactions,^64−69^ we have neglected them in the present study and will be addressed in future work.

At both M06-2X and NEVPT2 levels of theory, the whole system in the deoxygenated deoxyHc-O_2_ form (d_Cu–Cu_ = 4.8 Å) keeps the triplet multiplicity of the oxygen molecule with a typical d_O–O_ of free O_2_ (1.19 Å), while the singlet becomes the lowest-energy state for the oxygenated oxyHc is formed (d_Cu–Cu_ = 3.6 Å). In oxyHc, d_O–O_ increases from 1.19 to 1.47 Å, clearly suggesting the stabilization of the peroxide moiety. The NEVPT2 calculations predict a lower-energy barrier for O_2_ binding to the Cu_2_ center than DFT and CASSCF (see Figure S3a, SI) and an O_2_ binding energy of ∼−2.3 kcal/mol, which is much closer to the experimentally observed binding enthalpy for hemocyanin (−6.0 kcal/mol)^33,34^ than that predicted by DFT (∼−11.9 kcal/mol). A comprehensive comparison of our findings with previous theoretical studies is presented in Table S2 of the Supporting Information.

Energetics along the binding event from deoxyHc-O_2_ (d_Cu–Cu_ = 4.80 Å) and oxyHc (d_Cu–Cu_ = 3.60 Å) are shown in Figure 2b. Intermediate states along the binding path are named IN with *N* = 1, 2, 3, 4*n*, 4 (see Figure S3b, SI). At the M06-2X level of theory (Figure 2b, orange line), electron transfer (ET) from Cu_2_ to O_2_ leads to a high-energy superoxide (O_2_^–^) intermediate as the Cu–Cu distance decreases from 4.8 to 4.0 Å. This process is followed by an intersystem crossing (ISC) accompanied by structural reorganization of the Cu–Cu distance (from I3: T(4.0, 1.47) to I4: S(3.6, 1.35) in Figure 2b), and a rapid second ET stabilizes the peroxide species bound to the Cu_2_ center (from I4: S(3.6, 1.35) to oxyHc: S(3.6,1.47) in Figure 2b). Contrarily, NEVPT2 (Figure 2b, violet line) predicts an earlier ET toward a superoxide species already at d_Cu–Cu_ = 4.3 Å (I2 in Figure 2b), which is largely stabilized when the Cu–Cu distance decreases further to 4.0 Å and is much more stable than the DFT superoxide at such d_Cu–Cu_ (I3 in Figure 2b). With this approach, we also predict a low-energy barrier for the first ET (18.7 kcal/mol) than via DFT (23.4 kcal/mol), while the triplet–singlet ISC occurs by retaining the atom connectivity (from I3: T(4.0, 1.35) to I3: S(4.0, 1.35) in Figure 2b). This suggests an exothermic and rapid ISC, which is followed by a second ET requiring a minimal energy barrier (∼2 kcal/mol). Overall, both approaches, DFT and NEVPT2, agree on an exothermic and nearly simultaneous ET and ISC pathway,^24,33,34^ but the formation of a stable superoxide species, as detected spectroscopically,^63^ is exclusively identified by the multireference method.

**Figure 3:**
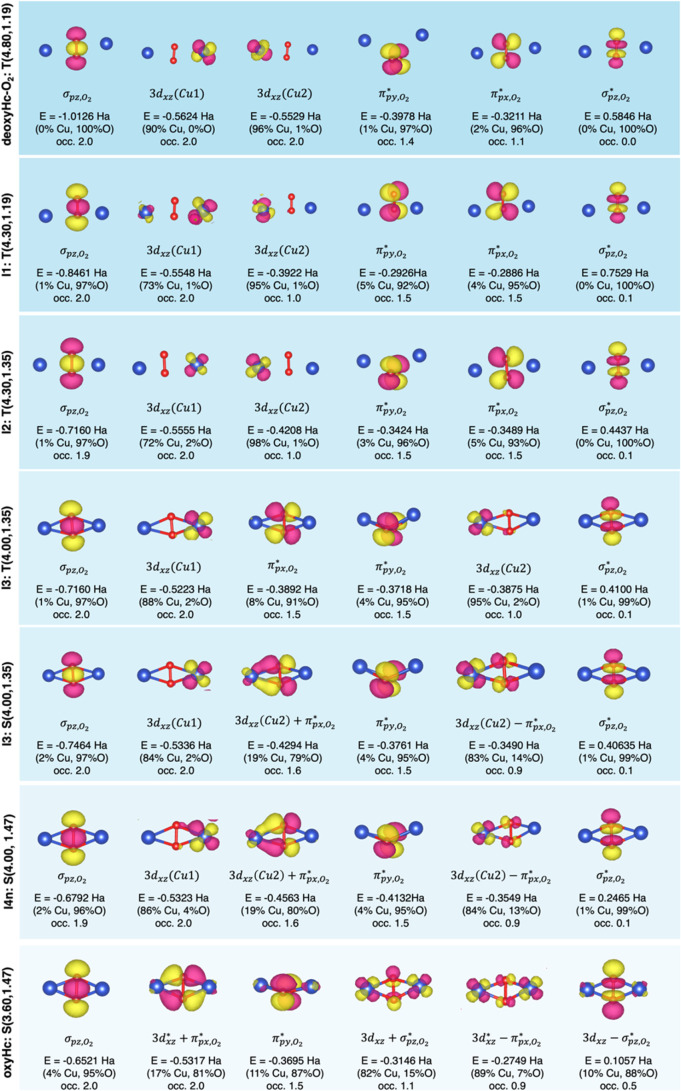
Significative MOs in the active space CAS(16e,12o) with their occupation numbers for the main point (d_Cu–Cu_, d_O–O_) along the oxygen-binding PES (see Figure 2b). MO energies and Hirshfeld MO population analysis are reported. Positive and negative isodensity surfaces (0.05 au) are depicted in yellow and magenta, respectively. Atomic color codes: Cu (blue) and O (red).

A detailed analysis of the key molecular orbitals (MOs) and electron distributions involved in O_2_ binding at the Cu_2_ active site (Figure 3), alongside the decreasing Löwdin bond order for O_2_ (see Table S3, SI), supports these transitions from oxygen to superoxide and then to peroxide. At I2: T(4.30, 1.35) and I3: T(4.00, 1.35), the fractional occupancy of the π* O_2_ MOs (approximately 1.5) supports the superoxide formation via the first ET from Cu to O_2_ at larger Cu–Cu distances. At the I3: S(4.00, 1.35) state, two distinct MOs arise from the overlap of Cu 3*d* orbitals with the O_2_ π* orbitals. The lower-energy bonding MO (Cu 3*d* + O_2_ π*) shows a partial occupancy of 1.5 electrons and contributions of 19% from Cu and 79% from O, indicating a predominant oxygen character. In contrast, the higher-energy antibonding MO (Cu 3*d* – O_2_ π*) is singly occupied and shows 83% Cu and 14% O contributions, highlighting a more copper-centric nature. These interactions give rise to a fully formed Cu–O_2_^–^ intermediate, where strong binding between the Cu hemocyanin core and the superoxide stabilizes the complex. The resulting singlet Cu–superoxide intermediate supports the fast intersystem crossing in the oxygen-binding mechanism.

The stable Cu–O_2_^–^ intermediate then undergoes a second electron transfer from Cu(II)/Cu(I) to superoxide. We can identify two possible pathways for the second ET at the NEVPT2 level of theory. In one case, the ET takes place as the Cu–Cu distance decreases from 4.0 Å to the oxyHc minimum at I4: S(3.6, 1.35). The Cu_2_O_2_ species then relaxes into a stable peroxide (O_2_^2–^) configuration, accompanied by an increase in the O–O bond length (from 1.35 to 1.47 Å). In the other, an elongation of the superoxide bond length from 1.35 to 1.47 Å (I4n: S(4.0, 1.47)) can occur before the Cu–Cu contraction and second ET. The electronic analysis confirms the superoxide and peroxo-like nature of the two transition states at S(4.00, 1.47) (Figure 3) and S(3.60, 1.35) (Figure S4, SI), respectively. While the S(4.00, 1.47) state holds the electronic structure, occupation, and Hirshfeld composition of the stable superoxide (I3: S(4.0, 1.35)), the S(3.60, 1.35) presents a further overlap with the second Cu atom of the Hc core and single occupied (Cu)_2_ 3*d* ± O_2_ π* MOs, as in the oxyHc (Figure 3). Therefore, the elongated superoxide pathway (I4n: S(4.00, 1.47)) is more favorable, with a low barrier of 1.7 kcal/mol compared to 3.2 kcal/mol as computed for the concerted mechanism (Figure 2b). These results denote a preference for further O–O bond elongation before significant structural reorganization in the hemocyanin active site, a process potentially relevant in the activity of ROS quenching (as a radical superoxide molecule) by deoxyHc.

Overall, the computed QM[NEVPT2]/MM energy profile suggests that the oxygen binding at the Hc dicopper core proceeds with low-energy barriers associated with both ET steps, delivering a binding energy closer to experimental data^33,34^ and a stable singlet superoxide intermediate not predicted by DFT. The superoxo formation is found to be the rate-limiting step, with peroxo species that could be produced with low energetic input. Indeed, the QM[NEVPT2]/MM energetics also agree with the mechanistic pathway of two nearly simultaneous ETs and ISC, as proposed in prior studies.^14,15,24^ Thus, we highlight the suitability of incorporating both static and dynamic correlation effects in describing the electronic transitions that govern oxygen binding at the hemocyanin active site.

### QM/MM Energetics of the Peroxo-to-Bis-μ-oxo Isomerization

**Figure 4:**
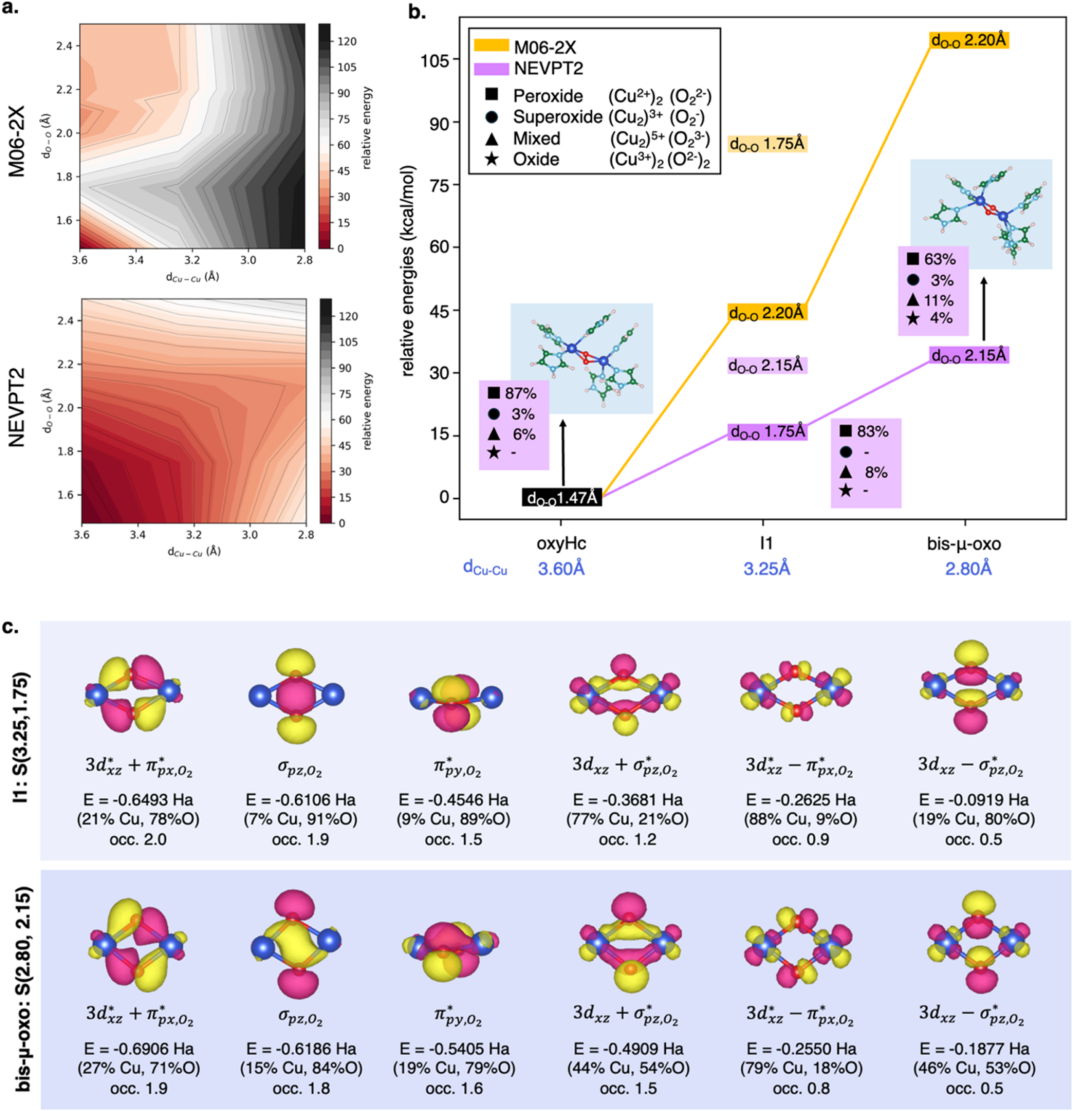
(a) Color energy maps of Hc peroxo-to-bis-μ-oxo isomerization computed at the QM[M06-2X]/MM and QM[NEVPT2]/MM levels of theory. (b) Energetics of oxyHc isomerization computed at QM[M06-2X]/MM (orange line) and QM[NEVPT2]/MM (violet line) levels of theory, with contributions of each possible configuration (peroxide (square), superoxide (circle), mixed (triangle), and oxide (star)) of the NEVPT2 wave function. Relative energies are referred to as oxyHc (*E*_S(3.6,1.47)_). Minimum energy structures of peroxo and bis-μ-oxo Hc are shown, while for the intermediate I1, see Figure S5b, SI. (c) Significative MOs in the active space CAS(16e,12o) with their occupation numbers for the main point (d_Cu–Cu_, d_O–O_) along the peroxo-to-bis-μ-oxo isomerization PES. MO energies and Hirshfeld MO population analysis are reported. Positive and negative isodensity surfaces (0.05 au) are depicted in yellow and magenta, respectively. Atomic color codes: Cu (blue) and O (red).

To gain deeper insights into the hemocyanin active site as an oxygen carrier, we extended our study to investigate the isomerization from a peroxo-to bis-μ-oxo species by analyzing (i) the O–O bond length ranging from 1.47 to 2.5 Å (d_O–O_ = 1.47, 1.75, 2.00, 2.10, 2.15, 2.20, 2.30, and 2.50 Å) and (ii) the Cu–Cu distance from 3.6 to 2.8 Å (d_Cu–Cu_ = 3.60, 3.25, and 2.80 Å), while we optimized the d_Cu–O_, which is always 2.0 Å. Since both peroxo and bis-μ-oxo configurations are stable in a singlet spin multiplicity and no intersystem crossings are expected, we only considered the singlet potential energy surfaces (S-PES). Figure 4a reports the PES for the peroxo-to-bis-μ-oxo isomerization, calculated at the QM[M06-2X]/MM (top panel) and QM[NEVPT2]/MM (bottom panel) levels of theory, while energetics along the isomerization event are shown in Figure 4b. Including both static and dynamic electronic correlations significantly lowers the isomerization energy barrier. Specifically, the NEVPT2 predicts energy barriers of 32.9 kcal/mol, slightly smaller than that obtained with CASSCF (57.8 kcal/mol; see Figure S5a, SI), and significantly lower than the 110.0 kcal/mol barrier obtained using DFT (M06-2X). However, in contrast to prior studies on small model clusters (see Table S2, SI),^45,46,48−50^ our multireference calculations stabilize the bis-μ-oxo state without altering the relative stability of the two minima. Such a discrepancy may stem from underestimating protein–ligand interactions in models that isolate the Cu_2_ core.

The low-energy barrier predicted by the NEVPT2 method arises from the gradual elongation of the O–O bond, which reaches a critical length of 2.15 Å as the Cu–Cu distance contracts to 2.8 Å in the bis-μ-oxo state (Figure 4b). In contrast, DFT calculations suggest a concerted reorganization of the O–O and Cu–Cu bond lengths. The stepwise isomerization pathway observed with NEVPT2 is further supported by the progressive weakening of the O–O bond and the corresponding strengthening of the Cu–O bonds (see Table S4, SI). This mechanistic distinction is corroborated by the electronic structural analysis (Figure 4c), which reveals an increasing overlap between the Cu *d*-orbitals and the O_2_ σ* molecular orbital along the reaction coordinate, alongside a partial population of the antibonding σ* orbitals of oxygen.

In the bis-μ-oxo state (S(2.80, 2.15)), bonding and antibonding Cu 3*d* ± O_2_ σ* MOs have almost equal Cu and O contributions, indicating a strong overlap between the two Cu 3*d* orbitals and the O_2_ σ* orbital. Moreover, we find that the bonding MO (Cu 3*d* + O_2_ σ*) is partially occupied with 1.5 electrons, while the antibonding MO (Cu 3*d* – O_2_ π*) has 0.5 occupancy. Such a strong overlap between the two Cu 3*d* orbitals with the O_2_ σ* MOs with partial occupation gives rise to a mixed configuration of such an isomer. As the system transitions to the bis-μ-oxo state, a gradual decrease in the occupation of MOs localized on the Cu atoms is observed, reflecting a diminished peroxide nature and a shift toward a mixed state, which is further corroborated by a small change in Mulliken charges from 0.87 in the peroxo state to 0.97 in the bis-μ-oxo state (Table S4, SI).

To gain deeper insights into electron redistribution along the isomerization pathway, analyzing the Hirshfeld population analysis of MOs and the NEVPT2 wave function, we identified the contributions of individual configurations, as detailed in Figure 4b. Notably, at the bis-μ-oxo minimum, the wave function is mainly (63%) of a peroxide nature ((Cu^2+^)_2_(O_2_^2–^)) and only 4% of an oxide ((Cu^3+^)_2_(O^2–^)_2_). Thus, we can confirm that the high multireference character of this bis-μ-oxo isomer with the Cu(III) configurations contributes marginally to the overall electronic structure. These findings align well with the previous analysis,^46^ suggesting that the bis-μ-oxo configuration in the hemocyanin active site likely represents a mixed-valence state of Cu atoms and not a fully further oxidation of the Hc dicopper core from Cu(II) to Cu(III).

Overall, we find that oxyHc predominantly exists in a peroxo state. Nevertheless, the low-energy barrier associated with the peroxo-to-bis-μ-oxo transition suggests that the bis-μ-oxo form may arise transiently as a local minimum, potentially during hemocyanin oxidative activity, such as in the quenching of ROS. Such an intermediate would subsequently rearrange back to the peroxo configuration, stabilizing the active site and enabling continued ROS mitigation. Thus, the bis-μ-oxo species, while transient, could play a role in the hemocyanin dynamic response to oxidative stress. In addition, our analysis supports the bis-μ-oxo state as primary peroxide in nature, with the hemocyanin environment allowing for a minor, yet significant, presence of mixed valency. This finding stresses the critical importance of advanced methods such as NEVPT2 in capturing the complex electronic and energetic details underlying the hemocyanin catalytic mechanism that simpler approaches, such as the standard DFT, often fail to resolve.

## Conclusions

This study reports a QM[NEVPT2]/MM investigation of the oxygen-binding and -isomerization mechanisms in hemocyanin, offering new insights into the enzyme functional reactivity. The optimized structures and binding energies for the deoxygenated and oxygenated Hc forms are in good agreement with the experimental data, confirming the validity of our QM/MM approach. The binding of O_2_ to the Hc dicopper core is characterized by a rapid, low-energy pathway with sequential electron-transfer (ET) steps, supported by the QM[NEVPT2]/MM results. The calculated O_2_ binding energies align more closely with experimental values when using NEVPT2 over DFT. Our findings identify the formation of a stable superoxide intermediate, consistent with previous experimental spectroscopic data,^70^ which results as the rate-limiting step in the oxygen-binding process. The QM[NEVPT2]/MM method effectively predicts a lower-energy barrier and a faster intersystem crossing (ISC) transition, allowing for the formation of a singlet peroxide species, consistent with previous studies.^14,15,24,33,34,70^ Analysis of the peroxo-to-bis-μ-oxo transition reveals a stepwise isomerization path, facilitated by the gradual elongation of the O–O bond rather than a concerted mechanism. This peroxo-to-bis-μ-oxo isomerization, while accessible, is energetically disfavored under physiological conditions with the peroxo form as the stable configuration for reversible oxygen binding. Nevertheless, the transient formation of the bis-μ-oxo intermediate, despite its higher-energy barrier, suggests that it may serve as a local minimum during Hc oxidative activity, facilitating ROS mitigation and stabilization of the active site. This transient state represents a possible key player in the dynamic response of Hc to oxidative stress.^13,42,71,72^

In conclusion, our study highlights the importance of multireference methods like NEVPT2 in capturing the intricate electronic transitions and mixed-valence states involved in the Hc catalytic mechanism. The new insights reinforce the need for advanced computational approaches to understand the subtle electronic and energetic features of metalloenzyme active sites, especially in biological redox processes.

## Supporting Information Available

Spin density of deoxyHc at the QM[M06-2X]/MM level of theory, validation of QM[NEVPT2]/MM (active spaces and singlet–triplet coupling), QM[CASSCF]/MM PES and minimum energy structures of intermediate states for the oxygen binding and isomerization paths, electronic analysis on the I4:S(3.60,1.35) state, Löwdin bond order and Mulliken charge analyses along the peroxo-to-bis-μ-oxo isomerization PES, and comparison of our findings with previous studies (PDF)
