# Supplementary material for: Revisiting Oxygen Transport Features of Hemocyanin with NEVPT2 Level QM/MM Calculations
Source: J Chem Theory Comput. 2025 Feb 6;21(4):2108–17. doi: 10.1021/acs.jctc.4c01668 (PMC11866748; doi:10.1021/acs.jctc.4c01668)
Supplement: Supplementary file 1 — ct4c01668_si_001.pdf [file ct4c01668_si_001.pdf]

SUPPORTING INFORMATION

Revisiting oxygen transport features of Hemocyanin with NEVPT2-level QM/MM calculations

Francesca Fasulo,[1] Aarón Terán More,[1] Michele Pavone,[2] Ana B. Muñoz-García[1*]

[1]*Department of Physics "E. Pancini", University of Naples Federico II, 80126 Napoli, Italy*

[2]*Department of Chemical Sciences, University of Naples Federico II, 80126 Napoli, Italy*

[*] ***Corresponding author***: *anabelen.munozgarcia@unina.it*

INDEX

S1. Spin density of deoxyHc (Cu_2[-O_2])..3

Figure S1. Spin density of deoxyHc (Cu_2[-O_2]) at QM[M06-2X]/MM level of theory. 3

S2. NEVPT2 validation: oxyHc active spaces CAS(ne,mo) and singlet-triplet splitting (-2J).........4

Figure S2. MOs considered in the different active spaces CAS(ne,mo) for oxyHc. 4

Table S1. Ground-state singlet-triplet splitting (-2J) of oxyHc at both QM[M06-2X]/MM and QM[CASSCF]/MM level of theory. 4

S3. Current results vs previous works 5

Table S2. Cu–Cu and O-O distances for Hc in deoxy, peroxo and bis-μ-oxo forms, energies of Hc oxygen-binding...5

S4. Oxygen binding at Hc active site. 6

Figure S3. (a) Color energy maps of oxygen binding at Hc active site computed at QM[CASSCF]/MM level of theory. Different spin multiplicity is declared. Relative energies are referred to deoxyHc ($E_{T(4.8,1.19)}$). (b) Minimum energy structures of each intermediate state (IN, N=1,2,3,4n,4) of the oxygen binding path in Fig. 2b. 6

Table S3. Cu–O and O-O Löwdin bond order (BO) for the main points (d_{Cu-Cu}, d_{O-O}) along oxygen binding PES (see Fig. 2b). 6

Figure S4. Significative MOs in the active space CAS(16e,12o) with their occupation numbers for the second electron transfer transition states (I4: S(3.60, 1.35) in Fig. 2b). MOs energies and Hirshfeld MOs population analysis are reported...6

S5. Peroxo-to-bis-μ-oxo isomerization. 7

Figure S5. (a) Color energy maps of Hc peroxo-to-bis-μ-oxo isomerization computed at QM[CASSCF]/MM level of theory. Relative energies are referred to oxyHc ($E_{S(3.6,1.47)}$). (b) Minimum energy structures of the intermediate state (I1) of the peroxo-to-bis-μ-oxo isomerization path in Fig. 4b. 7

Table S4. Cu–O and O-O Löwdin bond order (BO) and the Mulliken charges of Cu atoms (q(Cu)) for the main points (d_{Cu-Cu}, d_{O-O}) along peroxo-to-bis-μ-oxo isomerization PES 7

S6. References..8

S1. Spin density of deoxyHc ($Cu_2[-O_2]$)

deoxyHc-O_2: $Cu_2[-O_2]$

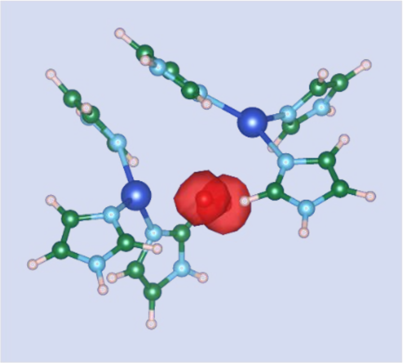

Figure S1. Spin density of deoxyHc ($Cu_2[-O_2]$) at QM[M06-2X]/MM level of theory. Isodensity surfaces (0.01 a.u.) are depicted as red and grey for positive and negative values, respectively. Atom color code: Cu (blue), N (light blue), C (green), H (light pink), O (red).

S2. NEVPT2 validation: oxyHc active spaces CAS(ne,mo) and singlet-triplet splitting (-2*J*).

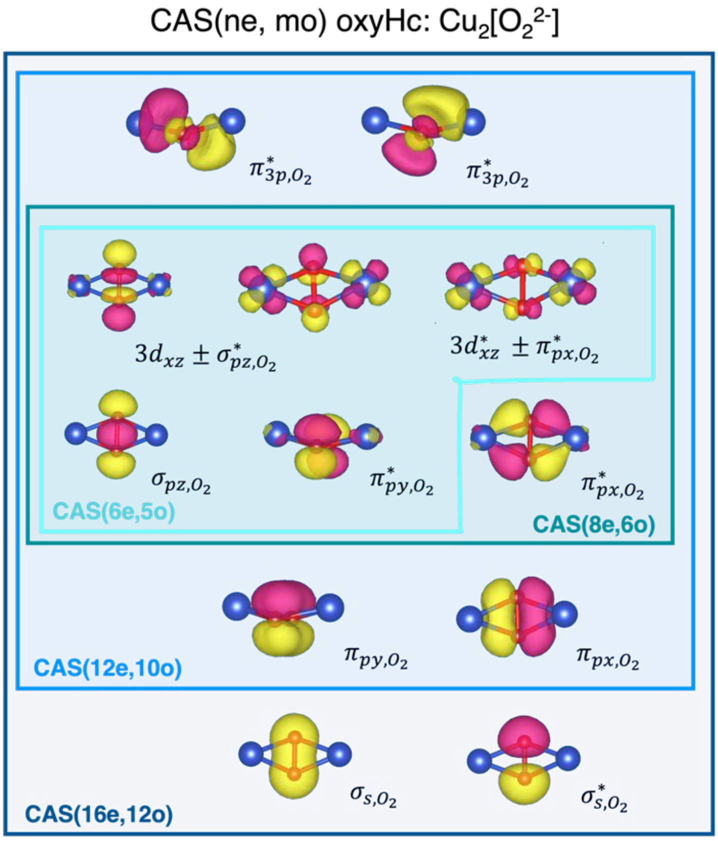

Figure S2. MOs considered in the different active spaces CAS(ne,mo) for oxyHc. Isodensity surfaces (0.05 a.u.) are depicted as yellow and magenta for positive and negative values, respectively. Atomic color code: Cu (blue), O (red).

Table S1. Ground-state singlet-triplet splitting (-2*J*) of oxyHc at both QM[M06-2X]/MM and QM[CASSCF]/MM level of theory. We computed the -2*J* values with the different active spaces CAS(ne,mo) in Fig. S2.

**QM[]/MM**	**M06-2X**	**CAS(6e,5o)**	**CAS(8e,6o)**	**CAS(12e,10o)**	**CAS(16e,12o)**
**-2*J* (cm^{-1})**	1085	543	531	680	690

S3. Current results vs previous works

Table S2. Cu–Cu and O-O distances for Hc in deoxy, peroxo and bis-μ-oxo forms, energies of Hc oxygen-binding (E_b) and peroxo-to-bis-μ-oxo isomerization (E_{isom}) in this work at QM[DFT]/MM and QM[NEVPT2]/MM levels of theory and in previous experimental[1–5] and theoretical works.[6–11]

	**this work QM(..)/MM**	**exp**	**other theor. works**
**Deoxy: d_{Cu-Cu} (Å)**	4.8 (M06-2X)	4.6 [1,2]	~ 4.80 (UBH&HLYP/MM)[6]
**Peroxo: d_{Cu-Cu} (Å)**	3.6 (M06-2X)	3.6 [1,2]	~3.40 (UBH&HLYP/MM)[6] ~ 3.5 (B3LYP)[7]
**Peroxo: d_{Cu-Cu} (Å)**	1.47 (M06-2X)	1.47 [1,2]	~ 1.47 (UBH&HLYP/MM)[6]
**E_b (kcal/mol)**	-11.9 (M06-2X)<br>-2.6 (NEVPT2)	-6.0 [3,4]	~ -15.6 (B3LYP)[7] ~ -18.9 (UBH&HLYP/MM)[6]<br>~ -16.5 (CASSCF)[8] ~ -27.9(CASPT2)[8]
**Peroxo: d_{Cu-Cu} (Å)**	2.8 (M06-2X)	2.8 [5]	2.86 (CASPT2) [9]
**Peroxo: d_{O-O} (Å)**	2.15 (M06-2X)	2.3 [5]	2.23 (CASPT2) [9] ~ 2.2 (PBE)[10]
**E_{isom} (kcal/mol)**	110.0 (M06-2X)<br>32.9 (NEVPT2)		~ 6-8(MRCI)[9] ~ -12 (CASPT2) [9]<br>~ 20 (B3LYP)[11] ~ -2.5(NEVPT2)[10]

S4. Oxygen binding at Hc active site.

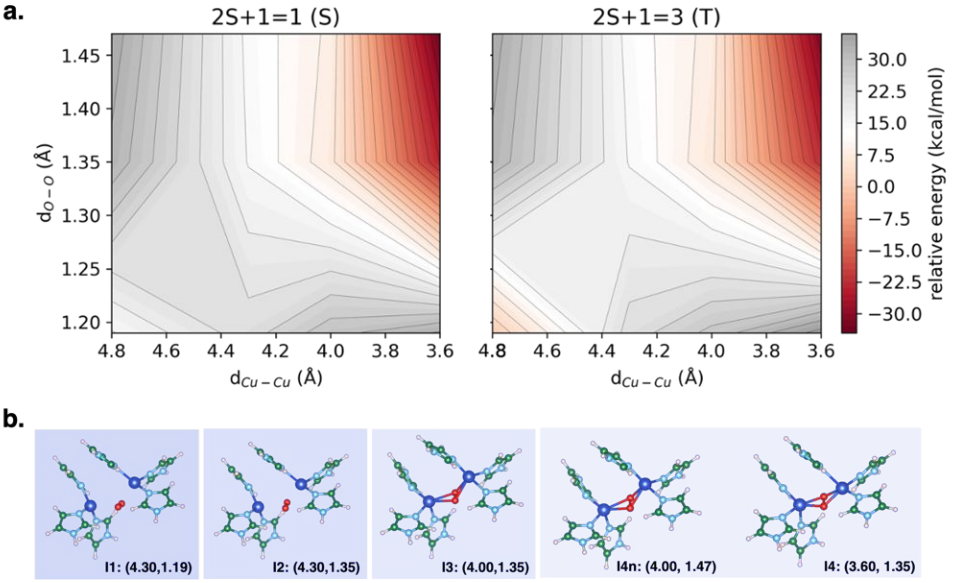

Figure S3. (a) Color energy maps of oxygen binding at Hc active site computed at QM[CASSCF]/MM level of theory. Different spin multiplicity is declared. Relative energies are referred to deoxyHc ($E_{T(4.8,1.19)}$). **(b)** Minimum energy structures of each intermediate state (IN, N=1,2,3,4n,4) of the oxygen binding path in Fig. 2b. Atomic color code: Cu (blue), N (light blue), C (green), H (light pink), O (red).

Table S3. Cu–O and O-O Löwdin bond order (BO) for the main points ($d_{Cu\text{-}Cu}$, $d_{O\text{-}O}$) along oxygen binding PES (see Fig. 2b).

**($d_{Cu\text{-}Cu}$, $d_{O\text{-}O}$)**	**(4.80,1.19)**	**(4.30,1.19)**	**(4.30,1.35)**	**(4.00,1.35)**	**(4.00,1.47)**	**(3.60,1.47)**
**BO(CuO)**	**0.1**	**0.2**	**0.2**	**0.3**	**0.3**	**0.6**
**BO(O_2)**	**1.9**	**1.9**	**1.6**	**1.6**	**1.3**	**1.1**

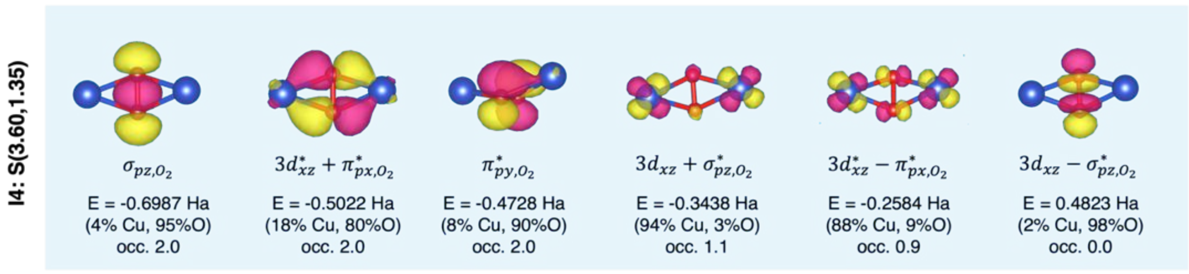

Figure S4. Significative MOs in the active space CAS(16e,12o) with their occupation numbers for the second electron transfer transition states (I4: S(3.60, 1.35) in Fig. 2b). MOs energies and Hirshfeld MOs population analysis are reported. Isodensity surfaces (0.05 a.u.) are depicted as yellow and magenta for positive and negative values, respectively. Atomic color code: Cu (blue), O (red).

S5. Peroxo-to-bis-μ-oxo isomerization.

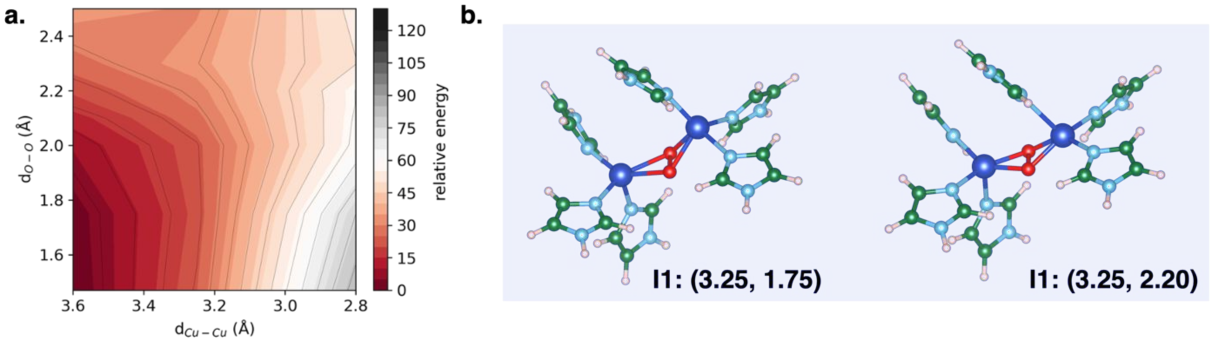

Figure S5. (a) Color energy maps of Hc peroxo-to-bis-μ-oxo isomerization computed at QM[CASSCF]/MM level of theory. Relative energies are referred to oxyHc ($E_{S(3.6,1.47)}$). **(b)** Minimum energy structures of the intermediate state (I1) of the peroxo-to-bis-μ-oxo isomerization path in Fig. 4b. Atomic color code: Cu (blue), N (light blue), C (green), H (light pink), O (red).

Table S4. Cu–O and O-O Löwdin bond order (BO) and the Mulliken charges of Cu atoms (q(Cu)) for the main points (d_{Cu-Cu}, d_{O-O}) along peroxo-to-bis-μ-oxo isomerization PES (see Fig. 4b).

**(d_{Cu-Cu}, d_{O-O})**	**S(3.60, 1.47)**	**S(3.25, 1.75)**	**S(2.80, 2.15)**
**BO(Cu-O)**	0.58	0.70	0.82
**BO(O-O)**	1.05	0.60	0.25
**q(Cu) (e^-)**	0.87	0.90	0.97

S6. References

(1) Hazes, B.; Kalk, K. H.; Hol, W. G. J.; Magnus, K. A.; Bonaventura, C.; Bonaventura, J.; Dauter, Z. Crystal Structure of Deoxygenated *Limulus Polyphemus* Subunit II Hemocyanin at 2.18 Å Resolution: Clues for a Mechanism for Allosteric Regulation. *Protein Sci.* **1993**, *2* (4), 597–619. https://doi.org/10.1002/pro.5560020411.

(2) Magnus, K. A.; Hazes, B.; Ton-That, H.; Bonaventura, C.; Bonaventura, J.; Hol, W. G. J. Crystallographic Analysis of Oxygenated and Deoxygenated States of Arthropod Hemocyanin Shows Unusual Differences. *Proteins Struct. Funct. Bioinforma.* **1994**, *19* (4), 302–309. https://doi.org/10.1002/prot.340190405.

(3) Klarman, A.; Daniel, E. Oxygen Binding Properties of Stripped (Calcium Ion and Magnesium Ion Free) Hemocyanin from the Scorpion Leirus Quinquestriatus. *Biochemistry* **1980**, *19* (23), 5176–5180. https://doi.org/10.1021/bi00564a004.

(4) Karlin, K. D.; Tolman, W. B.; Kaderli, S.; Zuberbühler, A. D. Kinetic and Thermodynamic Parameters of Copper-Dioxygen Interaction with Different Oxygen Binding Modes. *J. Mol. Catal. Chem.* **1997**, *117* (1–3), 215–222. https://doi.org/10.1016/S1381-1169(96)00250-6.

(5) Halfen, J. A.; Mahapatra, S.; Wilkinson, E. C.; Kaderli, S.; Young, V. G.; Que, L.; Zuberbühler, A. D.; Tolman, W. B. Reversible Cleavage and Formation of the Dioxygen O-O Bond Within a Dicopper Complex. *Science* **1996**, *271* (5254), 1397–1400. https://doi.org/10.1126/science.271.5254.1397.

(6) Saito, T.; Thiel, W. Quantum Mechanics/Molecular Mechanics Study of Oxygen Binding in Hemocyanin. *J. Phys. Chem. B* **2014**, *118* (19), 5034–5043. https://doi.org/10.1021/jp5003885.

(7) Metz, M.; Solomon, E. I. Dioxygen Binding to Deoxyhemocyanin: Electronic Structure and Mechanism of the Spin-Forbidden Two-Electron Reduction of O_2. *J. Am. Chem. Soc.* **2001**, *123* (21), 4938–4950. https://doi.org/10.1021/ja004166b.

(8) Bernardi, F.; Bottoni, A.; Casadio, R.; Fariselli, P.; Rigo, A. Ab Initio Study of the Mechanism of the Binding of Triplet O_2 to Hemocyanin. *Inorg. Chem.* **1996**, *35* (18), 5207–5212. https://doi.org/10.1021/ic960102j.

(9) Rode, M. F.; Werner, H.-J. Ab Initio Study of the O_2 Binding in Dicopper Complexes. *Theor. Chem. Acc.* **2005**, *114* (4–5), 309–317. https://doi.org/10.1007/s00214-005-0692-6.

(10) Varela Lambraño, R.; Vivas-Reyes, R.; Visbal, R.; Zapata-Rivera, J. Evaluation of the Electronic Structure and Charge Transfer in the $Cu_2O_2^{2+}$ Core Using Multiconfigurational Methods. *Theor. Chem. Acc.* **2020**, *139* (3), 56. https://doi.org/10.1007/s00214-020-2570-7.

(11) Cramer, C. J.; Włoch, M.; Piecuch, P.; Puzzarini, C.; Gagliardi, L. Theoretical Models on the Cu_2O_2 Torture Track: Mechanistic Implications for Oxytyrosinase and Small-Molecule Analogues. *J. Phys. Chem. A* **2006**, *110* (5), 1991–2004. https://doi.org/10.1021/jp056791e.
